# The Role of Parieto-Occipital Junction in the Interaction between Dorsal and Ventral Streams in Disparity-Defined Near and Far Space Processing

**DOI:** 10.1371/journal.pone.0151838

**Published:** 2016-03-21

**Authors:** Aijun Wang, You Li, Ming Zhang, Qi Chen

**Affiliations:** 1 Center for Studies of Psychological Application and School of Psychology, South China Normal University, Guangzhou 510631, China; 2 Guangdong Key Laboratory of Mental Health and Cognitive Science, South China Normal University, Guangzhou 510631, China; 3 Epilepsy Center, Guangdong 999 Brain Hospital, Guangzhou 510510, China; 4 Department of Psychology, Soochow University, Suzhou 215123, China; Centre de Neuroscience Cognitive, FRANCE

## Abstract

Neuropsychological and functional MRI data suggest that two functionally and anatomically dissociable streams of visual processing exist: a ventral perception-related stream and a dorsal action-related stream. However, relatively little is known about how the two streams interact in the intact brain during the production of adaptive behavior. Using functional MRI and a virtual three-dimensional paradigm, we aimed at examining whether the parieto-occipital junction (POJ) acts as an interface for the integration and processing of information between the dorsal and ventral streams in the near and far space processing. Virtual reality three-dimensional near and far space was defined by manipulating binocular disparity, with -68.76 arcmin crossed disparity for near space and +68.76 arcmin uncrossed disparity for near space. Our results showed that the POJ and bilateral superior occipital gyrus (SOG) showed relative increased activity when responded to targets presented in the near space than in the far space, which was independent of the retinotopic and perceived sizes of target. Furthermore, the POJ showed the enhanced functional connectivity with both the dorsal and ventral streams during the far space processing irrespective of target sizes, supporting that the POJ acts as an interface between the dorsal and ventral streams in disparity-defined near and far space processing. In contrast, the bilateral SOG showed the enhanced functional connectivity only with the ventral stream if retinotopic sizes of targets in the near and far spaces were matched, which suggested there was a functional dissociation between the POJ and bilateral SOG.

## Introduction

Visual information processing draws upon two anatomically distinct ‘streams’ of projections from the primary visual cortex (V1): A ventral stream projecting to the inferior temporal cortex and a dorsal stream projecting to the posterior parietal cortex [1]. The perception/action model of the ventral/dorsal stream function proposes that the ventral stream transforms visual information for the purpose of perception, while the dorsal stream processes visual information for the preparation and execution of actions [2–5]. Following this model, the ventral stream transforms visual information into perceptual representations, e.g., size, shape and color of objects. Such perceptual representations play an essential role in the identification of objects. In contrast, the dorsal stream transforms visual information into sensorimotor representations, e.g., the locations of objects. Such sensorimotor representations are crucial in visually guiding actions directed at those objects, such as manual reaching, pointing and grasping. Thus, the execution of a goal-directed action may depend on dedicated on-line sensorimotor representations in the dorsal stream, while the selection of appropriate target objects depends on the perceptual representations in the ventral stream. If we intend to accomplish a coherent behavior, the information in the ventral and dorsal streams must interface; therefore, a remaining central and critical issue is how the two streams interact in the intact brain during the production of adaptive behavior.

Electrophysiological and tract tracking studies in monkeys suggested role of visual area 6 (V6) complex as a potential candidate region in which information from the ventral and dorsal streams is integrated. The V6 complex is well-suited for this integrative function as it contains both visual and somatomotor cells [6–8]. Moreover, the V6 is anatomically located at the interface between the somatic and peripheral visual representations in the cerebral cortex: It is reciprocally and retinotopically interconnected with the striate visual cortex as well as with several posterior parietal areas [9–11]. In the human brain, the POJ is a putative homologue of monkey V6 complex [12–14]. Anatomically, the POJ is located in the ventral part of the anterior bank of the parieto-occipital sulcus, which matches the anatomical locus of the V6 [9, 14–18]. In addition, in a previous study, we found that the POJ is activated whenever perceptual representations in the ventral stream have to interact with sensorimotor representations in the dorsal stream [19]. Therefore, the existing evidences suggest that the POJ is a potential candidate region in which information from the ventral and dorsal visual streams is integrated.

The primary aim of the present study was to directly examine whether the POJ acts as an interface for the integration and processing of information between the dorsal and ventral streams. By adopting the virtual reality technique, we attempted to provide a three-dimensional space as the setting for a visual-manual task. We hypothesized that, in a visual-manual task, there were higher demands for the integration of information from two streams when a visual-manual task demands the processing of a visual target presented in the far space than in a visual-manual task that requires the processing of a visual target presented in the near space. Previous neuropsychological and imaging studies revealed that the dorsal and ventral streams differentially supported the near and far space processing [20–24]. Since no direct actions can be implemented in the far space, the far space processing differentially activates the ventral stream; in contrast, because an individual can directly act upon and manipulate objects in the near space, the dorsal stream subserves the near space processing [25, 26]. When targets of a visual-manual task were presented in the far space, on the one hand, the perceptual representation of far space targets depended on the ventral stream, on the other hand, according to the perception/action model of two visual streams, the sensorimotor representation of the manual action towards targets drew on the dorsal stream. Therefore, during far space processing, perceptual representations in the ventral stream have to be transformed into sensorimotor representations in the dorsal stream, and there were thus higher demands for the integration of information from the two streams to produce task relevant response.

Moreover, in three-dimensional space of the present study, stimuli in the near and far spaces could be either retinotopic size matched (same size in retina) or perceived size matched (same size in perception). If the retinotopic size were matched, bottom-up visual inputs between the near and far space processing could be equated, but participants would subjectively perceive larger objects in the far space. In contrast, if the perceived size were matched, the subjective perception could be equated, but the retinal inputs differed between the near and face spaces processing. However, previous studies mostly focused on the former and ignored the latter, which might confound the effect of near/far space with the stimulus size [27–29]. In order to accurately reveal the neural substrates respectively involved in the near and far space processing and to fully understand the functions of the POJ in spatial processing, the present study adopted both the ‘match’ (retinotopic size matched) and ‘natural’ (perceived size matched) conditions to present stimuli.

In the present functional MRI study, we directly addressed whether the interaction between the ventral and dorsal streams took place in the POJ (i.e., the human homologue of area V6). By employing virtual reality technique, we asked participants to perform tasks with a three-dimensional object presented either in the near or far space via stereo goggles, under both the ‘match’ and ‘natural’ conditions. Near and far space was defined by manipulating different binocular disparities, i.e. -68.76 arcmin crossed disparity for near space and +68.76 arcmin uncrossed disparity for near space. Since we were most interested in the differential processing between the near and far spaces, we contrasted the near and far space processing (i.e., the ‘Near > Far’ contrast) and then conjoined the ‘Near > Far’ contrasts for the ‘match’ and ‘natural’ conditions, thus creating a near or far space preference region that was independent of object size. Then, we performed psychophysiological interaction (PPI) analysis to investigate the functional connectivity between the resulting region (POJ) and the dorsal and ventral streams. With the neural activity in the POJ as the physiological factor, and the ‘Near > Far’ contrast as the psychological factor, we predicted that the POJ would show enhanced functional connectivity with both the dorsal and ventral streams during the far space processing.

## Materials and Methods

### Ethics Statement

Before the experiments, all participants gave their informed written consent in accordance with the Code of Ethics of the World Medical Association (Declaration of Helsinki), and the study protocol was approved by the Ethics Committee of the South China Normal University.

### Participants

Nineteen participants (mean age, 24 ± 3 years old; 7 male) took part in the present study. They were all right handed, and had normal or corrected to normal visual acuity. None of them had a history of neurological or psychiatric disorders.

### Apparatus, stimuli and experimental setup

A goggle-based MR-compatible system (VisuaStim Digital, Resonance Technologies, Northridge, California) provided two separate VGA with digital dual video inputs for stereoscopic display, each with a resolution of 800 (horizontal) × 600 (vertical) pixels at 60 Hz refresh rate. The horizontal extent of the field of view was 30 degree. The default viewing distance was 75 cm. The dual-display stereoscopic video, with 0.5 mega pixel resolution in a 0.25 square area, yielded 3D images by delivering slightly disparate images to each eye (binocular disparity). The 3D objects were generated by Blender (free open source 3D content creation software, http://www.blender.org), exported as Direct X files, and presented on a grey background by custom made Presentation scripts (Presentation Software package, Neurobehavioral Systems, Inc., Albany, CA).

**Fig 1:**
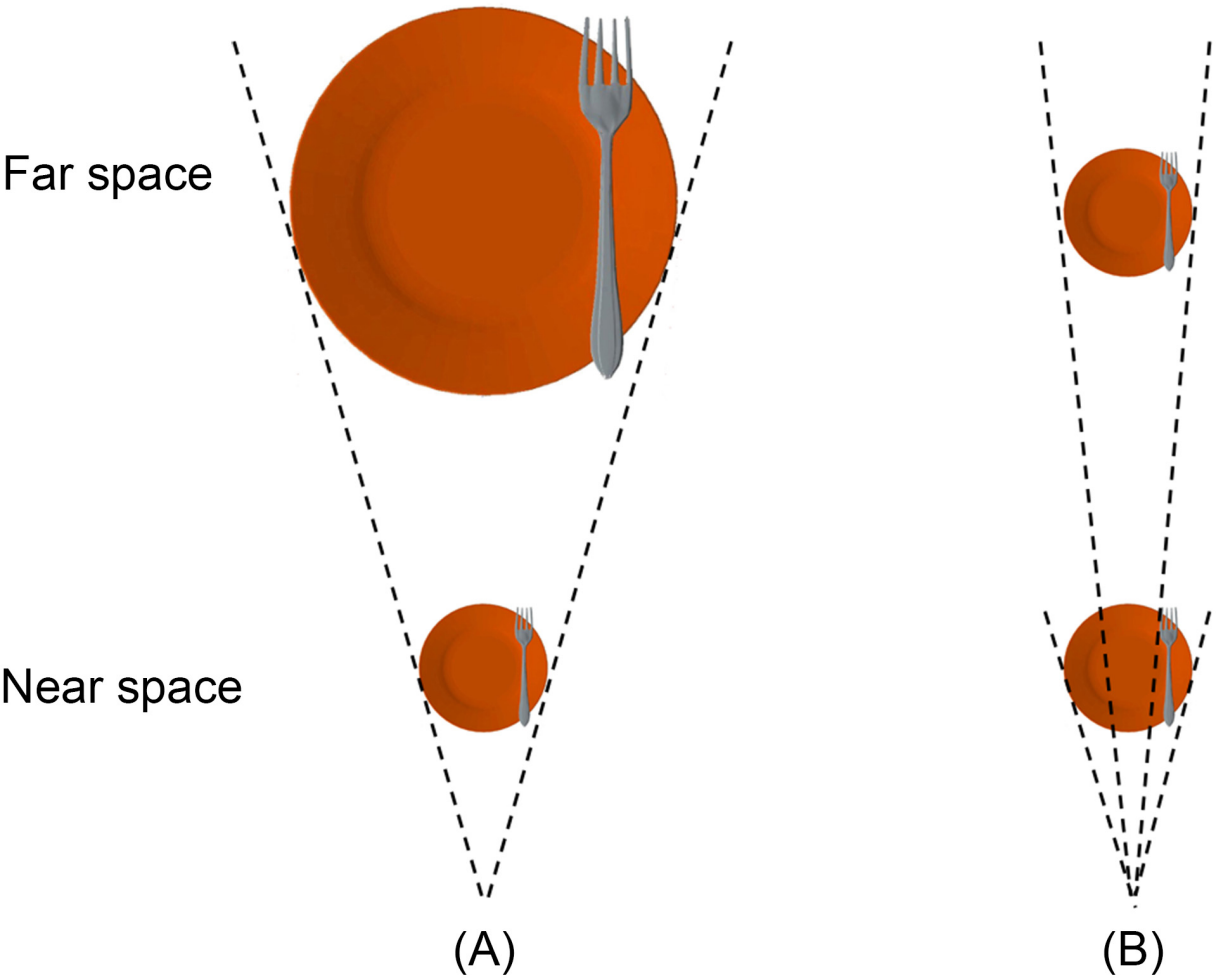
Experimental stimuli (top view). Three-dimensional stimuli consisted of a fork intersecting an plate either in the far space or in the near space both in ‘match’ (visual angles of stimuli were matched in the near and far spaces) stimuli (A) and ‘natural’ (visual angles of stimuli were same in the near and far spaces) stimuli (B). (Reprinted from [19] under a CC BY license, with permission from [Massachusetts Institute of Technology], original copyright [2012].).

In order to make the scenarios of the present study similar as in the real world, we used familiar but virtual tools which contained two 3D objects (Fig 1): A color fork on the top of a round orange plate, there were two possible luminance values for the color of the fork: 64, 64, 64 or 192, 192, 192 (24 bits RGB color coding). The diameter of the plate was 15° of visual angle, and the nearer end of the fork was 2.5° of visual angle. In each trial, these stimuli were presented either in the near or far space. In the near space condition, the 3D objects popped out of the default screen of the goggles, and the distance from the center of the plate to the participants’ eyes was 50 cm. For the far space condition, the objects appeared behind the default screen and were 150 cm away from the participants’ eyes. The different target distances were simulated by adjusting the binocular disparity. The binocular disparity between the near and far depth spaces was ± 68.76 min of arc relative to the fusion plane (zero disparity), which was 75 cm from the participants. These values are near the maximum disparity separation possible without loss of fusion since a practice experiment showed that trained observers had no trouble fusing the two images with a binocular disparity of ± 68.76 min of arc. Observers reported having a clear perception of the near and far depth spaces while keeping their gaze straightforward. The near and far spaces stimuli were presented at the same height (y = 0, at the level of the eyes) and tilted by 13° toward the participants.

### Experimental design and tasks

Each participant completed two runs: a run for ‘match’ stimuli (visual angles of stimuli were matched in the near and far spaces, e.g., the diameter of the plate was 15° of visual angle in the near space (50 cm), the diameter of the plate was 45° of visual angle in the far space (150 cm), see Fig 1A) and a run for ‘natural’ stimuli (visual angles of stimuli were same in the near and far spaces, e.g., the diameters of the plates were both 15° of visual angle in the near and far spaces, see Fig 1B). In both runs, blocked functional MRI design was adopted and there were two types of blocks: the near blocks in which all stimuli were presented in the near space; and the far blocks in which all stimuli were presented in the far space. As described in our previous study [19], participants were asked to perform three types of tasks on an identical stimulus set. In the allocentric (ALLO) task, participants were asked to judge whether the fork was on the left or right side of the plate. Participants were asked to press a button on the left or right side of a response pad. In the egocentric (EGO) task, participants were asked to judge whether the fork was on the left or right side of the midsagittal of their own body. Participants were asked to press a button on the left or right side of a response pad. In the high-level baseline (HLB) task (i.e., non-spatial luminance discrimination tasks), participants were asked to discriminate whether the fork had a high or low luminance. Participants were asked to press one button for high luminance and the other button for low luminance. The mapping between the levels of luminance and the response buttons was counterbalanced across participants. In the middle of scanning, an instruction (6 seconds) was displayed asking participants to switch hands. Nine participants switched from left hand to right hand in the middle of scanning, and vice versa for the other 10 participants. For both hands, participants were required to use their index fingers and middle fingers to press one of the two buttons on the left or the right side of the response pad. Before the functional MRI experiment, all participants completed a training session of 15 minutes with a slightly different setting (shutter glasses) outside the scanner to familiarize them with the tasks.

Thus, the experimental design was a two (stimulus size: ‘match’ vs. ‘natural’) by two (spatial domains: Near vs. Far) by three (tasks: ALLO, EGO, and HLB) blocked design. For each of ‘match’ and ‘natural’ runs, this design resulted in six experimental conditions: egocentric task in near space (‘Near_EGO’), allocentric task in near space (‘Near_ALLO’), high-level baseline task in near space (‘Near_HLB’), egocentric task in far space (‘Far_EGO’), allocentric task in far space (‘Far_ALLO’), and high-level baseline task in far space (‘Far_HLB’). Accordingly, for each run, participants alternated between six types of experimental blocks. Each block began with a 3 second visual instruction, informing participants about the type of task in the following block. The target presentation time during each trial was 150 ms, such a short stimulus-on time was chosen to minimize eye movements. Participants were required to keep their gaze straightforward and converge towards the frontal plane in which the near space or far space stimuli appeared during the near space blocks or far space blocks, respectively. The importance of not moving their eyes was repeatedly emphasized. The duration of each trial was 1650 ms. There were 10 trials per block, resulting in a block duration of 16.5 seconds. There were 8 repetitions for each of the 6 block types.

### Data acquisition and pre-processing

A 3T Siemens Trio system with a standard head coil was used to obtain T2*-weighted echo-planar images (EPI) with blood oxygenation level-dependent (BOLD) contrast. The matrix size was 64 × 64, and the voxel size: 3.1 × 3.1 × 3.0 mm^3^. Thirty-six transversal slices of 3 mm thickness that covered the whole brain were acquired sequentially with a 0.3 mm gap (repetition time = 2.2 sec, echo time = 30 msec, field of view = 220 mm, flip angle = 90°). There were two runs of functional scanning, each lasted for 16.5 minutes (450 EPI volumes). The first five volumes were discarded to allow for T1 equilibration effects. Additional high-resolution anatomical images were acquired using a standard T1-weighted 3D MP-RAGE sequence, the voxel size was 1 × 1 × 1 mm^3^.

Data were preprocessed with Statistical Parametric Mapping software SPM8 (Wellcome Department of Imaging Neuroscience, London, http://www.fil.ion.ucl.ac.uk). Images were realigned to the first volume to correct for interscan head movements. Then, the mean EPI image of each participant was computed and spatially normalized to the MNI single participant template using the ‘unified segmentation’ function in SPM8. This algorithm is based on a probabilistic framework that enables image registration, tissue classification, and bias correction to be combined within the same generative model. The resulting parameters of a discrete cosine transform, which define the deformation field necessary to move individual data into the space of the MNI tissue probability maps, were then combined with the deformation field transforming between the latter and the MNI single participant template. The ensuing deformation was subsequently applied to individual EPI volumes. All images were thus transformed into standard MNI space and re-sampled to 2 × 2 × 2 mm^3^ voxel size. The data were then smoothed with a Gaussian kernel of 8 mm full-width half-maximum to accommodate inter-participant anatomical variability.

### Statistical analysis of imaging data

Data were highpass-filtered at 1/128 Hz and analyzed with a general linear model (GLM) as implemented in SPM8. At the first level, ‘match’ and ‘natural’ runs were modeled separately. In each run, the GLM was used to construct a multiple regression design matrix that included two conditions: ‘Near’ and ‘Far’. Each condition was modeled by a boxcar reference vector (16.5 s) convolved with a canonical synthetic hemodynamic response function (HRF). Additionally, all the instructions and the six head movement parameters derived from the realignment procedure were included as covariates of no interest. Task blocks in which participants did not adhere to the task instructions (error rates within such blocks were above 60%), were separately modeled as another regressor of no interest. This occurred in two of the nineteen participants who each misperformed in one of a total of 48 task blocks. Parameter estimates were subsequently calculated for each voxel using weighted least squares to provide maximum likelihood estimators based on the temporal autocorrelation of the data. No global scaling was applied. For each participant, simple main effects for both of the two conditions were computed. The two first-level individual contrast images in both the ‘match’ and ‘natural’ runs were then fed into a within-subjects ANOVA at the second group level employing a random-effects model (flexible factorial design in SPM8 including an additional factor modeling the participant means). In the modeling of variance components, we allowed for violations of sphericity by modeling non-independence across parameter estimates from the same participant and allowing unequal variances both between conditions and participants using the standard implementation in SPM8. We were especially interested in the differences between the two conditions (‘Near > Far’ and ‘Far > Near’). Areas of activation were identified as significant only if they passed a conservative threshold of *p* < 0.001, corrected for multiple comparisons at the cluster level, with an underlying voxel level of *p* < 0.001 uncorrected [30]. For the conjunction analysis, the conjunction null hypothesis, instead of the global null hypothesis, was tested as implemented in SPM8 [31, 32].

### Psychophysiological interaction analysis (PPI)

We used the POJ and bilateral SOG (derived from the conjunction of contrast ‘Near > Far’ in the ‘match’ run ∩ ‘Near > Far’ contrast in the ‘natural’ run) as a source region to estimate the context-specific functional modulation of neural activity across the whole brain caused by neural activity in the POJ and bilateral SOG using psychophysiological interaction (PPI) analysis. PPI analysis allows for detecting regionally specific responses in one brain area in terms of the interaction between input from another brain region and a cognitive/sensory process [33]. We used the contrast ‘Near > Far’ as the psychological factor and used the neural activity in the POJ and bilateral SOG as the physiological factors (seeds), respectively. For each participant, the conjunction between the contrast ‘Near > Far’ from the ‘match’ run and the contrast ‘Near > Far’ from the ‘natural’ run was first calculated at the individual level. Subsequently, for neural activations in the above neural contrast, participant’s individual peak voxel was determined as the maximally activated voxel within a sphere of 16 mm radius (i.e., twice the smoothing kernel) around the coordinates of the peak voxel within the left SOG (MNI: -10, -86, 42), the right SOG (MNI: 22, -84, 38) and the POJ (MNI: -2, -78, 30) from the second level group analysis, respectively. Individual peak voxels from every participant were located in the same anatomical structure. Next, the POJ and bilateral SOG time series were extracted from a sphere of 4 mm radius (twice the voxel size) around the individual peak voxels (without deconvolution because of the block design). PPI analysis at the first individual level employed one regressor representing the extracted time series in the given region of interest (ROI) in the POJ and bilateral SOG (the physiological variable), one regressor representing the psychological variable of interest, that is ‘Near > Far’, and a third regressor representing the cross product of the previous two (the PPI interaction term). An SPM was calculated to reveal areas whose activation was predicted by the PPI interaction term, with the physiological and the psychological regressors treated as confounding variables, i.e., by putting 1 on the PPI regressor and 0 on the physiological and the psychological regressors, respectively. At the group level, random-effects analysis was adopted: the individual SPMs corresponding to the PPI term in each participant were subsequently entered into a one-sample t test (*p* < 0.001, FWE correction for multiple comparisons at cluster level with an underlying voxel threshold at *p* < 0.005, uncorrected).

## Results

### Main effect of spatial domain (‘Near > Far’)

**Fig 2:**
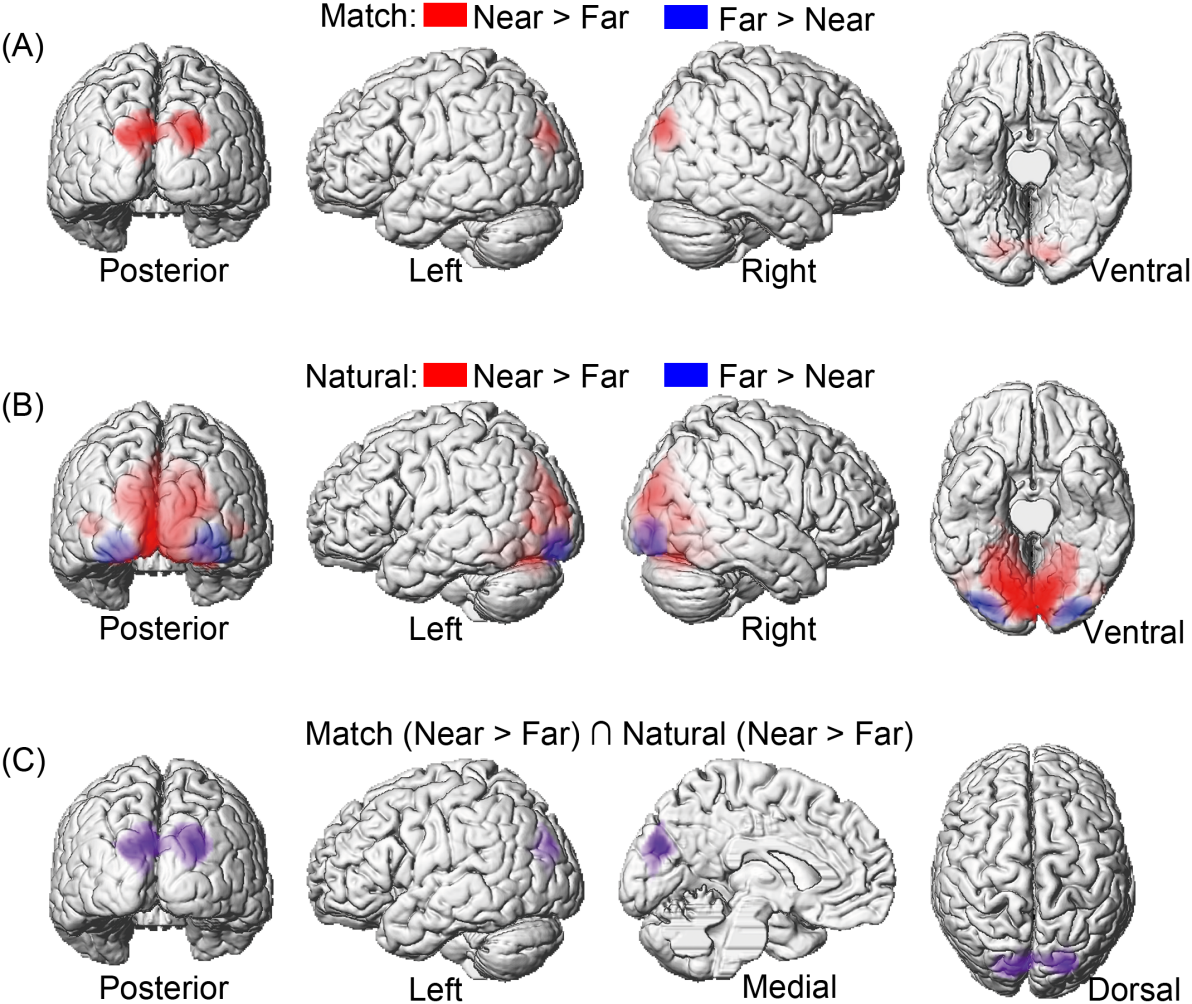
Main effect of spatial domain. (A) The bilateral SOG and the POJ showed significantly higher neural activity in the near space processing than in the far space processing (i.e., ‘Near > Far’) in the ‘match’ condition. No significant activation was found for the reverse contrast (i.e., ‘Far > Near’). (B) The primary visual cortex, the POJ and SOG showed significantly higher neural activity in the near space processing than in the far space processing (i.e., ‘Near > Far’) in the ‘natural’ condition (red regions). The bilateral inferior occipital gyrus (IOG) were activated for the reverse contrast (i.e., ‘Far > Near’). (C) Conjunction between the ‘Near > Far’ contrast in the ‘match’ and ‘natural’ conditions revealed the POJ and bilateral SOG.

We first identified brain regions that were associated with differential processing of spatial domains (both of the two conditions) in the ‘match’ run. We found that an extended activation cluster, including the bilateral SOG and the POJ, showed significantly higher neural activity in the near space processing than in the far space processing (i.e., ‘Near > Far’) (Fig 2A red regions and Table 1A). No significant activation was found for the reverse contrast (i.e., ‘Far > Near’).

**Table 1:** Brain regions showing significant relative increases of BOLD response associated with the different spatial domains (near and far space), and the conjunction contrast between the ‘match’ and ‘natural’ conditions.

Anatomical Region	Side	Cluster Peak (mm)	*t*-Score	*k*_E_ (voxels)
**(A)‘Match’ Condition: Near > Far**				
*Superior Occipital Gyrus*	*R*	*24*, *-90*, *30*	*7*.*73*	*1416*
	*L*	*-10*, *-88*, *36*	*7*.*04*	
*Parieto-Occipital Junction*	*L*	*-4*, *-80*, *30*	*3*.*77*	
**(B)‘Natural’ Condition: Near > Far**				
*Lingual*	*R*	*12*, *-80*, *-6*	*17*.*53*	*16678*
*Calcarine*	*L*	*-6*, *-88*, *2*	*16*.*63*	
*Superior Occipital Gyrus*	*R*	*22*, *-88*, *24*	*14*.*76*	
	*L*	*-12*, *-92*, *20*	*13*.*15*	
*Parieto-Occipital Junction*	*L*	*0*, *-74*, *52*	*4*.*61*	
**Far > Near**				
*Inferior Occipital Gyrus*	*L*	*-32*, *-92*, *-8*	*10*.*65*	*774*
	*R*	*36*, *-92*, *-2*	*10*.*04*	*873*
**(C) Conjunction: Near > Far**				
*Superior Occipital Gyrus*	*R*	*24*, *-88*,*30*	*7*.*63*	*1734*
	*L*	*-14*, *-86*, *30*	*5*.*80*	
*Parieto-Occipital Junction*	*L*	*-2*, *-78*, *30*	*4*.*50*	

The coordinates (x, y, z) correspond to MNI coordinates. Displayed are the coordinates of the maximally activated voxel within a significant cluster as well as the coordinates of relevant local maxima within the cluster (*in Italics*).

We also identified brain regions that were associated with differential processing of spatial domains in the ‘natural’ run. We found an extended activation cluster, including the right lingual gyrus, the left calcarine sulcus, the bilateral SOG, and the POJ, showed significantly higher neural activity in near space processing than in far space processing (i.e., ‘Near > Far’) (Fig 2B red regions and Table 1B). For the reverse contrast, the bilateral IOG was activated (i.e., ‘Far > Near’) (Fig 2B blue regions and Table 1B).

More importantly, we isolated the brain regions, which showed near space preference independent of object size. A conjunction analysis between the main effect of spatial domain (‘Near > Far’) in the ‘natural’ and ‘match’ runs was performed. The conjunction null hypothesis, instead of the global null hypothesis, was tested as implemented in SPM8 [31, 32]. The POJ and bilateral SOG were activated in the conjunction analysis (Fig 2C and Table 1C).

### Psychophysiological interaction (PPI) analysis with POJ as the source region

**Fig 3:**
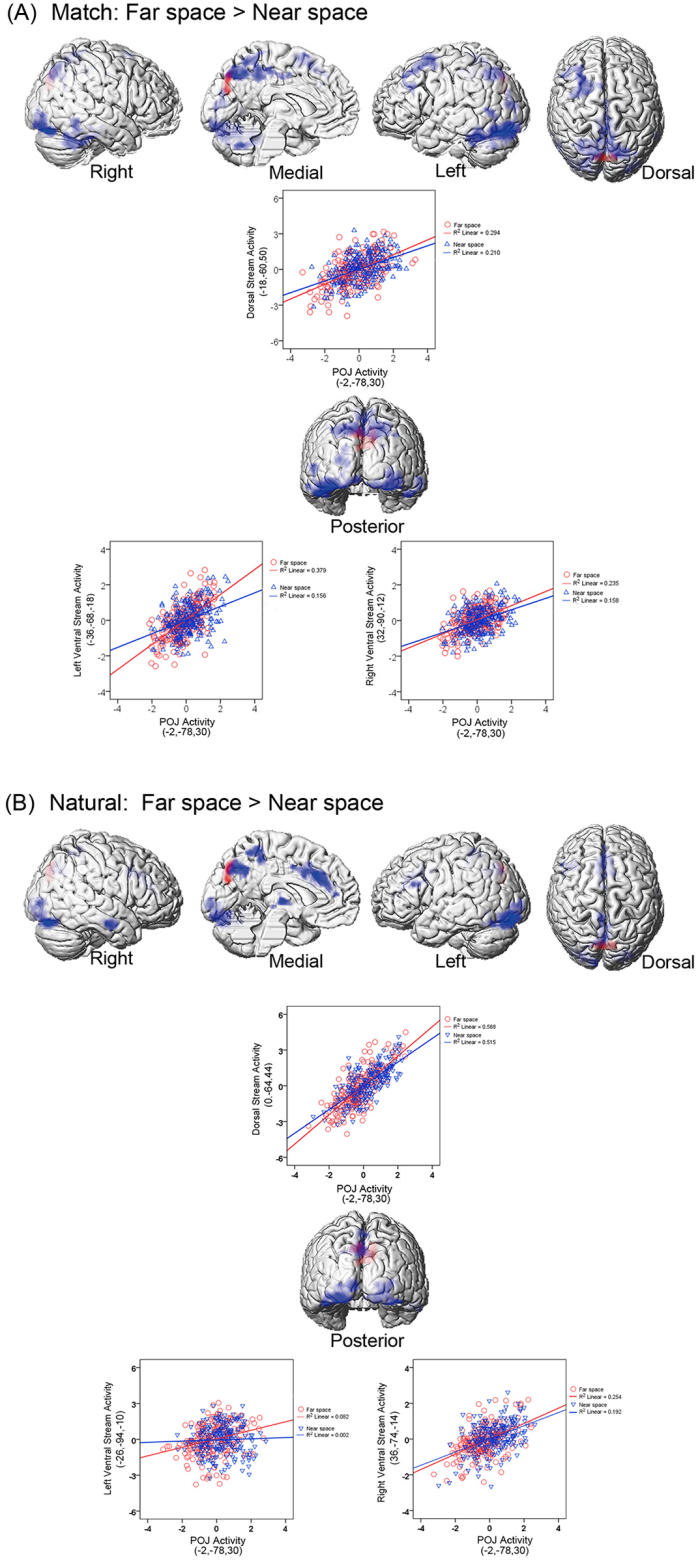
Psychophysiological interaction (PPI) analysis with the POJ as the source region and with the ‘Near > Far’ contrast as the psychological factor. The source region in the POJ is marked in red. (A) PPI activation in ‘match’ condition. Both the dorsal and ventral streams (blue) showed significant context-dependent co-variations with the neural activity in the POJ. The coupling was stronger in the far space than in the near space. In order to give a clearer view of ventral cortical structures, the cerebellum was removed in the ventral view display. PPI analysis based on the neural activity in the POJ (red) for a representative participants were shown. Mean corrected neural activity in the right inferior occipital gyrus, the left fusiform gyrus, the left inferior temporal gyrus, the left superior parietal gyrus and the right precuneus is displayed as a function of mean corrected activity in the POJ (i.e., first principal component from a sphere of 4 mm radius) in the near space (blue dots and lines) and far space (red dots and lines) blocks. (B) PPI activation in ‘natural’ condition. Both the dorsal and ventral streams (blue) showed significant context-dependent co-variations with the neural activity in the POJ. The coupling was stronger in the far space than in the near space. In order to give a clearer view of ventral cortical structures, the cerebellum was removed in the ventral view display. PPI analysis based on the neural activity in the POJ (red) for a representative participants were shown. Mean corrected neural activity in the left inferior occipital gyrus, the right fusiform gyrus, the right lingual gyrus, the right middle occipital gyrus, the left precuneus is displayed as a function of mean corrected activity in the POJ (i.e., first principal component from a sphere of 4 mm radius) in the near space (blue dots and lines) and far space (red dots and lines) blocks.

PPI analysis was performed with the main effect of spatial domain (i.e., ‘Near vs. Far’) as the psychological factor and with the neural activity in the POJ as the physiological factor. The POJ showed increased neural coupling with both the dorsal and ventral visual streams in the far space processing than in the near space processing. The ‘match’ and ‘natural’ runs revealed consistent results (see Fig 3 and Table 2). No significant modulation of neural coupling was obtained in the reverse direction (i.e., ‘Near > Far’). Please note that, PPI analysis calculated the coupling (correlation) between neural activity in different brain regions irrespective of the height of neural activity. Thereby, although the height of neural activity in the POJ was significantly higher in the near space than it was in the far space, the neural couplings between the POJ and both the ventral and dorsal streams were higher in the far space than they were in the near space.

**Table 2:** Brain regions that showed higher functional connectivity with the POJ in the Far blocks versus the Near blocks.

Anatomical Region	Side	Cluster Peak (mm)	*t*-Score	*k*_E_ (voxels)
**(A) ‘Match’ Condition**				
**Ventral visual stream**				
*Inferior occipital gyrus*	*R*	*32*,*-90*,*-12*	*7*.*84*	*2255*
*Fusiform gyrus*	*L*	*-34*,*-82*,*-16*	*7*.*67*	
*Inferior temporal gyrus*	*L*	*-48*,*-50*,*-20*	*4*.*76*	*299*
**Dorsal visual stream**				
*Superior parietal gyrus*	*L*	*-18*,*-60*,*50*	*6*.*70*	*440*
*Precuneus*	*R*	*6*,*-56*,*68*	*6*.*12*	
**(B) ‘Natural’ Condition**				
**Ventral visual stream**				
*Inferior occipital gyrus*	*L*	*-26*,*-94*,*-10*	*7*.*37*	*1435*
*Fusiform gyrus*	*R*	*36*,*-74*,*-14*	*6*.*06*	*994*
*Lingual gyrus*	*R*	*26*,*-86*,*-6*	*5*.*82*	
**Dorsal visual stream**				
*Middle occipital gyrus*	*R*	*26*,*-90*,*4*	*5*.*63*	*994*
*Precuneus*	*L*	*0*,*-64*,*44*	*6*.*04*	*502*

The coordinates (x, y, z) correspond to MNI coordinates. Displayed are the coordinates of the maximally activated voxel within a significant cluster as well as the coordinates of relevant local maxima within the cluster (*in Italics*).

### Psychophysiological interaction (PPI) analysis with bilateral SOG as the source region

**Fig 4:**
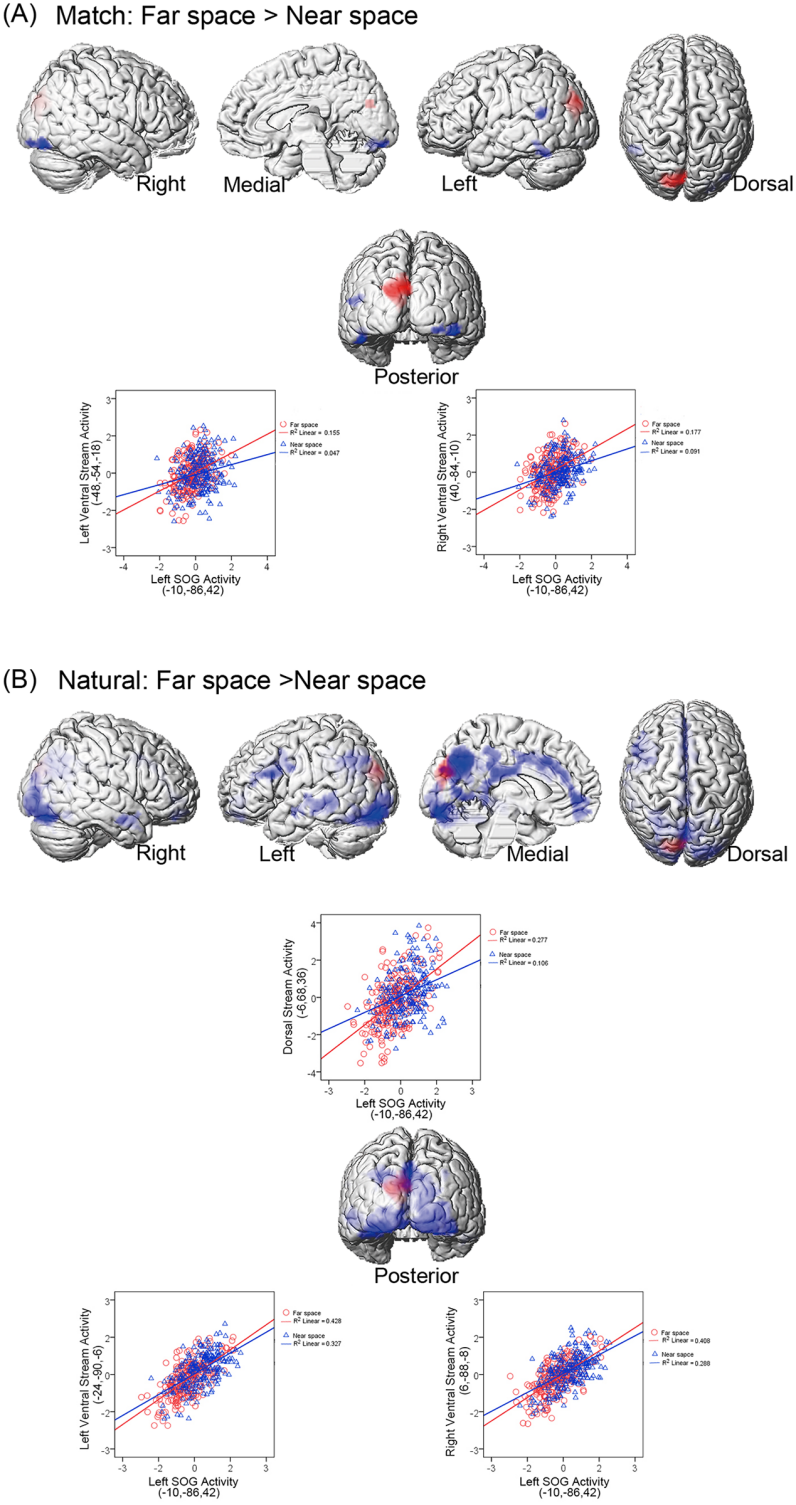
Psychophysiological interaction (PPI) analysis with left SOG as the source region and with the ‘Near > Far’ contrast as the psychological factor. The source region in the left SOG is marked in red. (A) PPI activation in ‘match’ condition. Only the ventral stream (blue) showed significant context-dependent co-variations with the neural activity in the SOG. The coupling was stronger in the far space than in the near space. In order to give a clearer view of ventral cortical structures, the cerebellum was removed in the ventral view display. PPI analysis based on the neural activity in the left SOG (red) for a representative participants were shown. A posterior view is given, with the cerebellum removed. Mean corrected neural activity in the right inferior occipital gyrus, the left fusiform, the left inferior temporal gyrus and the left middle temporal gyrus is displayed as a function of mean corrected activity in left SOG (i.e., first principal component from a sphere of 4 mm radius) in the near space (blue dots and lines) and far space (red dots and lines) blocks. (B) PPI activation in ‘natural’ condition. Both the dorsal and ventral streams (blue) showed significant context-dependent co-variations with the neural activity in the left SOG. The coupling was stronger in the far space than in the near space. In order to give a clearer view of ventral cortical structures, the cerebellum was removed in the ventral view display. PPI analysis based on the neural activity in the left SOG (red) for a representative participants were shown. Mean corrected neural activity in the left inferior occipital gyrus, the right lingual, the left middle temporal gyrus, the left middle cingulate gyrus, the left precuneus, the left inferior frontal gyrus and the left precentral gyrus is displayed as a function of mean corrected activity in left SOG (i.e., first principal component from a sphere of 4 mm radius) in the near space (blue dots and lines) and far space (red dots and lines) blocks.

PPI analysis was performed with the main effect of spatial domain (i.e., ‘Near vs. Far’) as the psychological factor and with the neural activity in the bilateral SOG as the physiological factor. For the ‘natural’ run, the bilateral SOG showed increased neural coupling with both the dorsal and ventral visual streams for far space processing than near space processing. The left and right SOG showed the same pattern, but connectivity was observed only between the left SOG and the dorsal and ventral visual streams (see Fig 4B and Table 3). However, for the ‘match’ run, the bilateral SOG showed increased neural coupling only with the ventral visual stream, connectivity was observed only showed the connectivity between the left SOG and the ventral visual stream, (see Fig 4B and Table 3).

**Table 3:** Brain regions that showed higher functional connectivity with the left SOG in Far blocks versus the Near blocks.

Anatomical Region	Side	Cluster Peak (mm)	*t*-Score	*k*_E_ (voxels)
**(A) ‘Match’ Condition**				
**Ventral visual stream**				
*Inferior occipital gyrus*	*R*	*40*,*-84*,*-10*	*5*.*94*	*647*
*Fusiform*	*L*	*-48*,*-54*,*-18*	*5*.*93*	*338*
*Inferior temporal gyrus*	*L*	*-44*,*-52*,*-10*	*5*.*08*	
*Middle temporal gyrus*	*L*	*-50*,*-56*,*22*	*5*.*78*	*344*
**(B) ‘Natural’ Condition**				
**Ventral visual stream**				
*Inferior occipital gyrus*	*L*	*-24*,*-90*,*-6*	*8*.*98*	*13653*
*Lingual*	*R*	*6*,*-88*,*-8*	*8*.*59*	
*Middle temporal gyrus*	*L*	*-56*,*-42*,*4*	*10*.*52*	*976*
**Dorsal visual stream**				
*Middle cingulate gyrus*	*L*	*-2*,*14*,*38*	*7*.*92*	*13653*
*Precuneus*	*L*	*-6*,*-68*,*36*	*7*.*65*	
*Inferior frontal gyrus*	*L*	*-54*,*14*,*28*	*6*.*73*	*1268*
*Precentral gyrus*	*L*	*-44*,*-2*,*34*	*5*.*80*	

The coordinates (x, y, z) correspond to MNI coordinates. Displayed are the coordinates of the maximally activated voxel within a significant cluster as well as the coordinates of relevant local maxima within the cluster (*in Italics*).

## Discussion

The present study directly examined whether the POJ acts as an interface for the integration and processing of information between the dorsal and ventral streams in disparity-defined near and far space processing. Although there was a variety of depth cue to implement depth perception, the present study defined near and far space by manipulating binocular disparities. Thus the observed effects of spatial domain were results of different binocular disparities. We first identified the brain regions that were associated with differential processing of spatial domains (near vs. far space). The POJ, along with the bilateral SOG, showed significantly higher neural activity in the near space processing than in the far space processing, independent of object size (Fig 2C and Table 1C). Anatomically, the POJ identified in the present study was located in the ventral part of the anterior bank of the parieto-occipital sulcus (Fig 2C), which matched the anatomical locus of the putative human V6. Functionally, the results of the present study were consisted with previous studies, which showed that the POJ was sensitive to stereoscopic cues and to the distance between object and observer [34–39]. For example, Quinlan and Culham (2007) used functional MRI to examine activation in the POJ as a function of viewing distance when multiple cues to target depth were available. The participants viewed disks presented at three distances (with equal retinal sizes). Results showed that the POJ demonstrated a near-space preference, with activation highest for near viewing, moderate for arm’s length viewing, and lowest for far viewing. In addition, Cardin and Smith (2011) used functional MRI to assess whether the brain combines disparity gradients with optic flow when encoding egomotion. Stereoscopic gradients were applied to expanding dot patterns presented to participants. The depth cues were either consistent with egomotion or inconsistent. Results showed that V6 responded well to all optic flow patterns but much more strongly when they were paired with consistent rather than inconsistent or zero disparity gradients. In the present study, binocular disparities of the near and far spaces were both consistent with those in real life. By conjoining the ‘Near > Far’ contrasts for the ‘match’ and ‘natural’ conditions, our results showed that the POJ was preferentially recruited in the near space processing, irrespectively of whether the objects sizes were perceptually or retinotopically same.

Second, and most pertinent to the aim of the present study, our PPI results found that the POJ showed enhanced functional connectivity with both the dorsal and ventral streams during the far space processing, independent of the retinotopic or perceived size of the targets, which lent support to the proposal that the POJ acted as an interface for the integration and processing of information between the dorsal and ventral visual streams. The areas showing enhanced functional connectivity in the PPI analysis overlapped with the areas of the dorsal and ventral visual streams identified in previous studies [1–3, 19, 25, 26]. It has been long proposed that the dorsal stream projecting from the striate cortex to the posterior parietal region mediates the required sensorimotor transformations for visually guided actions directed at such objects, while the ventral stream of projections from the striate cortex to the infero-temporal cortex plays the major role in the perceptual identification of objects [1–3]. Neuroimaging study also showed that manual bisection task activated the dorsal visual stream, including the extrastriate, superior parietal, and precuneus cortex, while perceptual bisection judgments activated the ventral visual stream, including the temporal cortex, extrastriate, inferior parietal cortex, anterior cingulate, and right dorsolateral prefrontal cortex [26].

Three-dimensional spatial processing of targets in the near reachable space depended on sensorimotor representations in the dorsal stream; in contrast, processing of targets that placed in the far space outside the distance of an outreached hand depended on perceptual representations in the ventral stream [23, 25, 26]. Therefore, when participants were demanded to make response to the far space targets, there was necessity to transform perceptual representations in the ventral stream into sensorimotor representations in the dorsal stream. Such transformation depended on the POJ acts as the interface for the exchange of information between the dorsal and ventral streams, which was indicated by the enhanced functional connectivity between the POJ and the two streams during the far space processing. Previous evidences suggested that tools enable human beings (and some primates) to manipulate objects that would otherwise not be reachable by hands, probably by transforming perceptual representations of objects in the far space to high level sensorimotor representations [20, 40–44]. The interface function of the POJ might underlie such remapping of the far space into the near space, in which vision and action are integrated, so that it was possible to reach and manipulate objects in the far space as if they were in the near space. Moreover, please note that, when looking at the height of neural activity, activation analysis highlighted the preferential role of the POJ in processing the near space stimuli (see Fig 2). However, with our PPI results taken into consideration, in the far space processing, although the neural activity of the POJ was not elevated, it played another role as interface that transformed perceptual and sensorimotor representations between the ventral and dorsal streams via enhanced neural coupling with the two streams (see Fig 3). Thus, our results revealed the two-sided functions of the POJ in three-dimension spatial processing: In the near space processing which depended solely on the sensorimotor representations in dorsal visual stream, the POJ acted as the locus to process the near space stimuli and to guide actions toward targets; while in the far space processing which needed the perceptual representations in the ventral stream to be transformed into sensorimotor representations in the dorsal stream, the POJ served as the interface in which information from the two visual streams exchanged.

Moreover, the PPI results in the present study demonstrated functional dissociation between the POJ and SOG. Previous studies usually defined the combination of the POJ and SOG regions as the superior parieto-occipital cortex (sPOC), which included the regions on either the posterior or anterior side of the superior parieto-occipital sulcus [36, 45–49]. Similarly, in the present study, when compared the height of neural activity between the near and far space processing, the POJ and SOG were activated as an extensive cluster showing similar psychological function. However, our PPI results indicate that the mechanisms of the POJ and SOG differed as to their functional connectivity with the dorsal and ventral streams. While the POJ showed functional connectivity with both the dorsal and ventral streams in both the ‘natural’ (perceived size matched) and ‘match’ conditions (retinotopic size matched), the SOG only showed functional connectivity with the ventral stream in the ‘match’ condition, yet showed functional connectivity with both the dorsal and ventral streams in the ‘natural’ condition. Some evidences indicated both the POJ and SOG played a critical role in planning and executing hand actions in the specific spatial location of the target in three-dimensional space [36, 46, 50–54]. However, other evidence showed that the SOG was activated during object perception [55]. Therefore, one tempting speculation was that the SOG might have a preference for perceptual presentation, rather than sensorimotor presentation.

In the present study, the different target distances of near and far spaces were simulated by adjusting the binocular disparity. Therefore, the differential effects of near and far spatial domains on the neural activity level and neural coupling were resulted from binocular disparity depth cue. Please note, the perception of depth or distance arises from a variety of depth cues: monocular cues, such as perspective, and binocular cues, such as disparity. Among all depth cues, disparity is the most frequently used to define spatial domain (i.e. the near and far extrapersonal spaces) in human [19, 56, 57] and other primates [58]. However, besides binocular disparity, other depth cue alone could also indexed near and far spaces. For example, Quinlan and Culham (2007) found that, in the absence of binocular disparity and monocular cues, the near preference of POJ could be still elicited by oculomotor cues (i.e. convergence, accommodation, and pupil size). In the second experiment of Quinlan and Culham’s, participants simply verged their eyes to maintain fixation on a small spot of light at one of the three distances. Results showed that the POJ displayed a gradient of activation to distances (Near > Medium > Far), which indicated that the near preference in the POJ might reflect the status of oculomotor cues. To complement the conclusion of Quinlan and Culham, the results of the present study indicated that, on the other side, near preference in the POJ could be elicited by binocular disparity alone, in the absence of oculomotor. Further investigation is needed to examine whether the differential representations of spatial domain in the brain were specific to particular depth cues (e.g. binocular disparity) or a property that general for all depth cues (e.g. monocular depth cues).

Taken together, by manipulating binocular disparity as depth cue, the present study found that, in addition to its role in the near space processing, the POJ showed enhanced functional connectivity with both the dorsal and ventral streams during the far space processing irrespective of the target size, which directly supports that the POJ acts as a neural interface between the dorsal and ventral streams in disparity-defined near space and far space processing.
